# Janus kinase inhibitors in autoimmune bullous diseases

**DOI:** 10.3389/fimmu.2023.1220887

**Published:** 2023-07-10

**Authors:** Dawei Huang, Yuexin Zhang, Luyang Kong, Jiajing Lu, Yuling Shi

**Affiliations:** ^1^ Department of Dermatology, Shanghai Skin Disease Hospital, Tongji University School of Medicine, Shanghai, China; ^2^ Institute of Psoriasis, Tongji University School of Medicine, Shanghai, China

**Keywords:** autoimmune bullous disease, Janus kinase inhibitor, pemphigus, pemphigoid, JAK-STAT signaling pathway

## Abstract

Autoimmune bullous disease (AIBD) is a severe skin disorder caused by autoantibodies that target intercellular or cell-matrix adhesion proteins. Currently, the preferred treatment for AIBD involves the use of glucocorticoids or traditional immunosuppressants. Additionally, the utilization of biological agents such as rituximab, omalizumab, and dupilumab is on the rise. However, effectively managing AIBD remains a challenge. The Janus kinase/signal transducers and activators of transcription (JAK/STAT) pathway has been implicated in various inflammatory diseases. In recent years, a range of drugs known as JAK inhibitors, which target this pathway, have been developed. Several studies have explored the efficacy and safety of JAK inhibitors for treating AIBD. Consequently, this review begins by examining the role of the JAK/STAT pathway in AIBD, summarizing the application of different JAK inhibitors in AIBD treatment, and emphasizing the importance of disease management in treating AIBD with JAK inhibitors. Furthermore, it highlights the need for a better understanding of the JAK/STAT pathway’s role in AIBD, as well as the effectiveness and safety of JAK inhibitors for treating this disease.

## 1 Introduction

Autoimmune bullous diseases (AIBDs) are rare conditions characterized by erosion, blistering, and bullous lesions on the skin and mucous membranes. These diseases arise from the production of autoantibodies that target proteins responsible for maintaining cell-to-cell and cell-to-matrix adhesion (1, 2). AIBDs can be categorized into two groups: pemphigus disease (intraepidermal immunobullous disease) and pemphigoid disease (subepidermal immunobullous disease). Pemphigus diseases encompass pemphigus vulgaris (PV), pemphigus foliaceus (PF), paraneoplastic pemphigus (PNP), immunoglobulin A (IgA) pemphigus, and others. Pemphigoid diseases include bullous pemphigoid (BP), epidermolysis bullosa acquisita (EBA), anti-lamininγ1 pemphigoid, mucous membrane pemphigoid (MMP), dermatitis herpetiformis (DH), linear IgA bullous dermatosis, pemphigoid gestationis, lichen planus pemphigoid (LPP), and bullous systemic lupus erythematosus (BSLE).

Since the availability of glucocorticoids and, later, non-steroidal immunosuppressive drugs, from the late 1960s, the mortality rate of AIBD patients has significantly decreased. However, as critical dermatological diseases, AIBDs are still associated with a high mortality rate (3, 4). In recent years, treatment modalities have gradually shifted toward more precise and targeted immune suppression and/or immune regulation. In particular, immunotherapy with biologics, such as rituximab, dupilumab, and omalizumab, is being clinically applied to treat various types of AIBDs with relatively good efficacy (5–9). Nevertheless, despite the availability of various treatment options, including glucocorticoids, traditional immunosuppressive drugs, and biological reagents, some patients still exhibit poor treatment responses or experience serious drug-related complications. Hence, there is a need for new therapeutic approaches that offer improved efficacy and fewer adverse effects (AEs).

The Janus kinase/signal transducers and activators of the transcription (JAK/STAT) pathway is vital for immune regulation, cell differentiation, apoptosis, and proliferation, affecting various cytokines and growth factors (10). This signaling pathway is associated with the pathophysiology of several autoimmune and autoinflammatory diseases. Therefore, blocking the JAK/STAT pathway has become an attractive approach for treating these diseases. In recent years, a variety of JAK pathway inhibitors have been used for the treatment of skin diseases such as psoriasis (11), alopecia areata (12), and atopic dermatitis (13). Since the JAK/STAT pathway is thought to play a role in the development of AIBDs, inhibiting this pathway could be a promising therapeutic strategy for these diseases.

This review aims to provide a concise overview of the potential involvement of the JAK/STAT pathway in the development of AIBDs and list the reported JAK inhibitors that have been used to treat AIBD. This information will serve as a foundation for further research on the pathogenesis, clinical diagnosis, and treatment of AIBDs.

## 2 The JAK/STAT pathway

To the best of our knowledge, only JAK inhibitors have been reported for the treatment of AIBD, while STAT inhibitors have not been similarly reported. Therefore, we will just summarize the function of JAK proteins. The JAK family comprises four proteins called TYK2, JAK1, JAK2, and JAK3 (14). JAK-dependent cytokine receptors transmit signals through various JAKs (**Table 1**), with each receptor having multiple subunits associated with JAK. A primary function of protein kinases is to transfer phosphate groups from guanosine triphosphate or adenosine triphosphate to amino acid hydroxyl groups (15). This fundamental mechanism is essential for receptors that lack intrinsic enzyme activity to facilitate biological processes. As a non-receptor tyrosine protein kinase, JAK can also conduct signal transduction through this mechanism. Typically, cytokine binding to their receptors triggers inflammatory signals. Receptors of type I and type II cytokines, including interferon (IFN), interleukin (IL)-2, IL-6, IL-12, IL-23, etc., are intrinsically deficient in enzyme activity and highly dependent on JAK for signal transduction (16). The JAKs initiate signaling pathways in the cell membrane that ultimately reach the nucleus after cytokines bind to their receptors (17). As part of this reaction, type I and II cytokine receptors undergo oligomerization to recruit JAKs and phosphorylate tyrosine residues, including tyrosine residues within the receptor chain. STAT proteins are then recruited, regulating the expression of related genes, resulting in antibody production, lymphocyte differentiation, increased inflammation, blister formation, and other pathophysiological processes.

**Table 1:** Summary of the cytokines associated with different JAK proteins.

JAKs	Cytokines/hormones
JAK1	IL-2, IL-4, IL-6 IL-7, IL-9, IL-10, IL-11, IL-13, IL-15, IL-19, IL-20, IL-21, IL-22, IL-24, IL-27, IL-28, IL-29, IFN-α, IFN-β, IFN-γ
JAK2	IL-3, IL-5, IL-6, IL-10, IL-11, IL-12, IL-13, IL-19, IL-20, IL-22, IL-23, IL-27, G-CSF, GM-CSF, GH, EPO, TPO, Leptin
JAK3	IL-2, IL-4, IL-7, IL-9, IL-13, IL-15, IL-21
TYK2	IL-6, IL-10, IL-11, IL-12, IL-13, IL-19, IL-20, IL-22, IL-27, IL-28, IL-29, IFN-α, IFN-β, IFN-γ

JAK, Janus kinase; IL, interleukin; IFN, interferon; GH, growth hormone; GM-CSF, granulocyte-macrophage colony stimulating factor; G-CSF, granulocyte-colony stimulating factor; EPO, Erythropoietin; TPO, thrombopoietin; TYK, tyrosine kinase.

## 3 The JAK/STAT pathway in AIBD

Multiple immune mechanisms, including cellular and humoral immunity, contribute to the development of pemphigus diseases. Researches have demonstrated that serum levels of tumor necrosis factor-α (TNF-α) and several type I and II cytokines, such as IL-1β, IL-4, IL-6, IL-8, IL-10, IL-12, and IL-15, are increased in patients with PV, while levels of IFN-γ are decreased (18–20). Furthermore, desmoglein (Dsg)3-reactive helper T cell (Th)1 and Th2 cells are found in patients with pemphigus disease. The presence of immunoglobulin (Ig)G4 and IgG1 antibodies against Dsg3 is directly related to the ratio of Dsg3 reactive Th1/Th2 cells (21). Thus, both Th1 and Th2 cells play an integral role in the pathogenesis of PV. Th1 cells overexpress IFN-γ to further mediate the immune response, while IL4, as a Th2-derived cytokine, regulates immunoglobulin conversion and antibody production by stimulating B cell proliferation (22). Cytokines such as IFN and IL-4 have been shown to play a biological role through JAK proteins. JAK1, JAK3, STAT6, and IL-4 signaling pathways are also important for the differentiation and proliferation of helper T cells (18). These discoveries suggest that the JAK/STAT signaling pathway plays a significant role in the pathogenesis of pemphigus diseases.

BP, a subepidermal form of AIBDs, is associated with autoantibodies targeting hemidesmosome components BP180 and BP230 (1). Studies using immunohistochemistry and western blotting analysis have shown that the expression of JAK/STAT proteins is significantly higher in BP-associated skin lesions compared to surrounding skin and healthy individuals (23). Furthermore, an increasing number of studies have demonstrated that Th2 cells play an important role in the production of antibodies in BP. It is worth noting that the chemotactic attraction of the Th2-like cytokine IL-4 towards eosinophils is very strong. IL-4 also takes part in the maturation and functional activity of eosinophils, as well as antibody production and the autoimmune response to BP (24, 25). The role of IL-17 in BP progression is evidenced by its upregulation of proteases involved in blister formation such as matrix metalloproteinase 9 and neutrophil elastase (26). The JAK signaling pathway has been proven to be closely related to the function of IL-4 and IL-17 (27, 28), which warrants further exploration of the role of the JAK signaling pathway in BP. Additionally, many JAK-dependent cytokines such as IL-6, IL-8, and IL-23 have been implicated in BP (29).

The role of JAK in other pemphigoid diseases, such as DH, MMP, and EBA, is similar to that observed for BP. For example, in DH and MMP, the expression of JAK/STAT-related proteins in the skin lesions of patients was significantly elevated (23). In the case of DH, eosinophilin and Th2 cytokines, such as IL-13, IL-4, and IL-5, also play an important role in the disease development (30). Additionally, a decrease in Treg cells and IL-10 has been observed in DH lesions (31), and the IL-31 concentration differs between healthy individuals and those with DH (32). This change in IL-31 concentration may be related to the intense itching typically associated with DH. In MMP, IL-4 and IL-13 not only affect disease onset but also influence the function of conjunctival fibroblasts, regulate scar formation, and thus, affect the prognosis of ocular MMP (33, 34). MMP is also associated with an increased localization of Th17 lymphocytes in lesions, especially in the conjunctiva, as well as local overexpression of IL-6, IL-12, and IL-17 (35). In EBA, high levels of IL-1β, IL-2, IL-6, IL-10, IL-21, TNF-β, and IFN-γ have been detected in serum samples (36). Similar to BP, in EBA, the Th2 pathway plays a key role in pathogenesis and itching. Consequently, the Th2 signaling pathway has received increasing attention in studies on pemphigoid diseases. Regarding treatment, an increasing number of reports have shown that the IL-4R monoclonal antibody dupilumab exhibits good efficacy in treating pemphigoid diseases (37). In addition to IL-4, the JAK/STAT pathway plays an important role in the function of other Th2 cytokines. In other words, inhibiting the JAK/STAT pathway can affect multiple Th2 cytokines. Therefore, evaluating the role of JAK/STAT in AIBDs is important for further research on pathogenesis and the development of therapeutic targets.

Animal models play a crucial role in understanding the mechanism of JAK inhibitors in treating AIBDs. However, there is a lack of research specifically using JAK inhibitors to treat AIBDs in mouse models, despite the existence of numerous reports on AIBDs in mouse models induced by antibody transfer, lymphocyte transfer, and immunization (38, 39). Interestingly, in veterinary medicine, oclacitinib, a selective JAK1 inhibitor for dogs, has shown promise in treating AIBDs like PF (40, 41). These observations suggest that JAK inhibitors may be beneficial for animals with AIBDs. It is still necessary to conduct animal model experiments focusing on AIBDs to gain further insights into the role of JAK inhibitors, representing an important avenue for future research.

## 4 Application of JAK inhibitors in AIBDs

### 4.1 Baricitinib

Baricitinib, which belongs to the class of JAK inhibitors, functions by blocking the JAK1 and JAK2-STAT signaling pathways. Additionally, it also inhibits the activity of IL-6, IL-12, and IL-23, thereby suppressing the differentiation of pathogenic Th17 cells. Baricitinib is used for treating rheumatoid arthritis, alopecia areata, and COVID-19, and has also been recommended for the treatment of psoriasis and other inflammatory-mediated diseases due to its considerable efficacy in controlling exaggerated inflammatory responses (42). Furthermore, some researchers have explored the potential of baricitinib as an off-label treatment for AIBDs (**Table 2**).

**Table 2:** The use of baricitinib in treating autoimmune bullous disease.

	Age/ gender	Types of AIBD	Complica-tion	Previous therapies	Treatment	Efficacy of initial use of JAK inhibitors	Tapering of JAK inhibitors	Efficacy of maintenance treatment	AE	Relapse
Sarny et al., 2018 (43)	43/M	MMP	Psoriasis	MTX, CP, MMF, RTX, IVIG, ADM, PSL	Baricitinib 4 mg/d, MTX 25mg/w, PSL 6mg/d	The disease was improved within two months	Baricitinib was stopped due to decreasing of neutrophils for 2 weeks. Subsequently, baricitinib was taken each day.	Progression of end-stage MMP seemed stopped.	None	NA
Burningham et al., 2022 (44)	69/F	MMP	T2DM, breast cancer	TCS, MTX, prednisone, IVIG, RTX, MMF, CP.	Baricitinib 2 mg/d, prednisone, MTX 12.5 mg/w, then increased to 20 mg/w.	Conditions of ocular, oral and esophageal were improved.	NA	The patient continued to improved.	NA	NA.
Xiao et al., 2022 (45)	83/M	BP	Psoriasis, stage III HT, postoperative lung cancer	Compound glycyrrhizin, TCS	Baricitinib 4 mg/d, Halometasone cream 0.5g/d	Skin lesions and pruritus improved significantly.	Baricitinib was halved and continued for 12 weeks	Both bullous and psoriatic lesions were in complete remission	None	A few new blisters were noted but faded away 3 days later.
Moussa et al., 2022 (46)	36/M	LPP	NA	TCS, SCS, MTX, AZP, ciclosporin, doxycycline, PUVA	Baricitinib 6.8 mg/d.	A significant improvement in pruritus and LPP lesions.	Baricitinib reduced to 3.4 mg/d	LPP almost completely relieved	NA	NA

AIBD, autoimmune bullous disease; JAKi, janus kinase inhibitors; AE, adverse effect; MMP, mucous membrane pemphigoid; BP, bullous pemphigoid; LPP, Lichen planus pemphigoides; M, male; F, female; MTX, methotrexate; CP, cyclophosphamide; MMF, mycophenolate mofetil; AZP, azathioprine; RTX, rituximab; IVIG, intravenous immunoglobulin; ADM, Adalimumab; PSL, prednisolone; TCS, topical corticosteroids; SCS, systemic corticosteroids; PUVA, psoralen plus ultraviolet A; T2DM, type 2 diabetes mellitus; HT, hypertension; NA, not available; mg/d, mg/day; mg/w, mg/week.

Baricitinib was initially utilized for the treatment of MMP in individual cases reported by Sarny et al. (43) and Burningham et al. (44). According to their reports, recalcitrant MMP was successfully managed with systemic glucocorticoids, methotrexate, and baricitinib. The favorable side effects of baricitinib and its oral administration make it a promising alternative to current interventions for MMP. Nevertheless, prospective studies are necessary to assess baricitinib’s effectiveness and establish its position within the treatment approach for MMP.

In the case of BP, Xiao et al. first reported a case of aggressive BP alongside plaque psoriasis that was effectively managed using baricitinib (45). The co-existence of psoriasis and the poor health conditions of the patient made the use of systematic glucocorticoids difficult, so baricitinib was chosen as an alternative. This report underscores the potential of baricitinib as a promising alternative treatment for co-existing plaque psoriasis and BP, or either condition individually.

Moussa et al. successfully treated a case of refractory LPP with baricitinib, highlighting its potential as an effective therapy for persistent cases (46). The limited availability of treatment options for LPP makes baricitinib a valuable addition. Concerning the underlying mechanism, Shao et al. discovered that in LPP, cytotoxic T-cell-mediated injury in keratinocytes was dependent on JAK2 and STAT1 signaling, and was inhibited by the JAK1/2 inhibitor baricitinib (47). These findings suggest that baricitinib may be able to alleviate the lichenoid tissue reaction in LPP.

One case report described a patient with epidermolysis bullosa pruriginosa (EBP) who had severe skin lesions and intense itching. Treatment with baricitinib resulted in a marked improvement in the patient’s condition (48).

### 4.2 Tofacitinib

Tofacitinib, an oral inhibitor targeting JAK1/3, has received U.S. Food and Drug Administration (FDA) approval for treating moderate-to-severe rheumatoid arthritis, active psoriatic arthritis, and ulcerative colitis in adult patients (49), as well as polyarticular juvenile idiopathic arthritis in children. *In vitro* studies have indicated that tofacitinib is capable of reducing TNF, IL-1β, and type I IFN production in dendritic cells derived from monocytes stimulated with antigenic lipopolysaccharide (50). Further, the use of tofacitinib for treating DH, MMP, EBA, BP, and PV has been reported in the literature, as described below (**Table 3**).

**Table 3:** The use of tofacitinib in treating autoimmune bullous disease.

	Age/ gender	Types of AIBD	Complication	Previous therapies	Treatment	Efficacy of initial JAK inhibitors	Tapering of JAK inhibitors	Efficacy of maintenance treatment	AE	Relapse
Kahn et al., 2021 (51)	76/M	DH	Celiac disease, HT	Dapsone, SAS	Tofacitinib 10mg/d	Significant improvement in pruritus, existing lesions and new lesions.	NA	Significant improved.	None	NA
James et al., 2021 (52)	79/F	MMP	HT, hyperlipidemia	MTX, MMF, RTX, CP, IVIG	Tofacitinib 11 mg/d, IVIG	Significant improvement in ocular inflammation.	NA	Free of further conjunctival inflammation.	None	NA
James et al., 2021 (52)	70/M	MMP	T2DM, myocardial infarction with subsequent stenting	Erythromycin ophthalmic ointment, 0.05% cyclosporine ophthalmic emulsion, MTX, MMF, CP, RTX	Tofacitinib 11 mg/d, MMF 2g/d	Ocular inflammation, nasal and oral ulcers resolved.	Stopped tofacitinib for over a month due to cost.	Rash recurred after tofacitinib withdrawal and improved after another oral dose.	None	Relapse due to withdrawal of medication
Fan et al., 2023 (53)	58/M	EBA	Pneumocystosis pneumonia, osteoporosis	PSL, MTX, dapsone, TCS	Tofacitinib 10 mg/d, PSL 8 mg/d	The disease was improved within one month.	Tofacitinib was tapered to 5 mg/d.	Blisters and erosions were almost disappeared.	None	None
Youssef et al., 2022 (54)	65/F	BP	Post-arthroplasty of the right patellar tendon rupture, hypothyroidism, hypercholesterolemia, HT, obesity, osteoarthritis, seronegative spondyloarthropathy	Prednisone, doxycycline, niacinamide.	Tofacitinib 20mg/d	BP dramatically improved.	Tofacitinib was cut to 10 mg/d due to sinus pain.	After tapering, she presented with recurrent pruritus and put tofacitinib back on 20 mg/d.	Sinus pain	Recurrent pruritus occurred due to the halving of dose.
Youssef et al., 2022 (54)	76/M	BP	Degenerative disc disease, steroid-induced atrial fibrillation	Prednisone, mycophenolate, dupilumab, RTX	Tofacitinib 20mg/d, dupilumab	Skin and pruritus were complete clearance.	NA	NA	NA	NA
Vander et al., 2022 (55)	34/F	PV	UC, controlled prolactinoma	Prednisone, topical clobetasol	Tofacitinib 10mg/d, RTX	Both paronychia and the quality of life have improved.	NA	NA	NA	NA

AIBD, autoimmune bullous disease; AE, adverse effect; DH, dermatitis herpetiformis; MMP, mucous membrane pemphigoid; EBA, epidermolysis bullosa acquisita; BP, bullous pemphigoid; PV, pemphigus vulgaris; M, male; F, female; TCS, topical corticosteroids; SCS, systemic corticosteroids; SAS, sulfasalazine; MTX, methotrexate; MMF, mycophenolate mofetil; RTX, rituximab; CP, cyclophosphamide; IVIG, intravenous immunoglobulin; PSL, prednisolone; HT, hypertension; T2DM, type 2 diabetes mellitus; UC, ulcerative colitis; NA, not available; mg/d, mg/day; mg/w, mg/week.

Tofacitinib was initially used for DH that did not respond to conventional treatment. Kahn et al. presented a DH patient treated with tofacitinib, observing notable clinical improvement and inhibition of new lesion development (51). These findings suggest that tofacitinib could be a potentially effective alternative for managing DH in patients who are unable to adhere to a gluten-free diet or have contraindications to dapsone, or in cases where these approaches prove unsuccessful.

Tofacitinib has also shown promising results as a treatment for MMP, as reported in some cases (52). Two patients suffering from ocular MMP had failed to respond to several therapies and were treated with tofacitinib, resulting in long-term control of conjunctival inflammation and no observed progression of sub-conjunctival fibrosis.

Fan et al. described a recurrent EBA patient that responded well to tofacitinib treatment (53). In this patient, they observed a decrease in circulating neutrophil counts, although their association with the clinical response is debated, as well as the anti-COL-7 IgG titer. These observations indicate the potential therapeutic value of JAK inhibitors for EBA.

With regard to BP, Youssef et al. found that tofacitinib was effective in treating two cases of BP, achieving treatment goals while avoiding the side effects of standard therapies, and improving itch control (54). This study represents the initial report highlighting the benefits of oral tofacitinib in the management of BP. Fan et al. subsequently published a case series that supported these findings (56), reporting that all patients were relieved of itching after one week of tofacitinib treatment, and levels of serum autoantibodies, eosinophils, IL-6, IL-17, and TNF-α were lower after tofacitinib administration compared to before. These findings indicate that tofacitinib has good therapeutic prospects in BP.

In 2018, a review indicated that tofacitinib could potentially serve as an alternative treatment for pemphigus diseases (57). It was suggested that both systemic and topical tofacitinib could have positive therapeutic effects on pemphigus diseases. However, determining the ideal dosage requires further exploration through clinical trials. Compared to rituximab, tofacitinib offers the advantage of being available in both oral and topical forms, whereas rituximab can only be administered intravenously. Furthermore, tofacitinib may be more efficacious than rituximab because it targets both T cells and B cells, while rituximab mainly affects B cells. In 2022, Vander et al. reported a case of a female patient presenting with mild to moderate PV with nail involvement (55). The combination of oral tofacitinib and rituximab infusions resulted in a strikingly rapid improvement in her nail symptoms. Although rituximab has been shown to achieve long-lasting remission in pemphigus patients, its onset of action is slow. Therefore, tofacitinib may have contributed to the swift symptom improvement in the Vander et al. case report. The case findings suggest that combining tofacitinib with rituximab could potentially lead to rapid disease improvement and long-term remission.

The treatment of EBP with tofacitinib has also been reported (58), and it has been found to inhibit the inflammatory response, relieve pruritus, and decrease the recurrent onset.

### 4.3 Ruxolitinib

Ruxolitinib is an oral JAK1/2 inhibitor approved for the treatment of polycythemia vera, myelofibrosis, vitiligo, and steroid-refractory graft-versus-host disease (SR-GVHD). It has shown efficacy in treating dermatologic diseases like psoriasis and alopecia areata as an oral or topical agent. In SR-GVHD, ruxolitinib is used as a salvage therapy, particularly for cases with oral involvement and bronchiolitis obliterans (BO). This is because ruxolitinib plays a critical role in inflammation and T-cell activation (59). Some studies report high efficacy and survival rates with ruxolitinib for treating SR-GVHD. Based on the similarities in pathogenesis and clinical features of PNP and GVHD, ruxolitinib was considered a potential option for managing persistent stomatitis and BO in a female PNP patient (60) (**Table 4**). Despite the patient experiencing consistent healing of the skin with prednisolone, azithromycin, and cyclosporine, there was no noticeable improvement in oral lesions and respiratory function until ruxolitinib was added. The potent immunosuppressive and anti-inflammatory activities of JAK inhibitors were considered valuable for managing BO in this refractory PNP case.

**Table 4:** The use of ruxolitinib and upadacitinib in treating autoimmune bullous disease.

	Age/ gender	Types of AIBD	Complica-tion	Previous therapies	Treatment	Efficacy of initial JAK inhibitors	Tapering of JAK inhibitors	Efficacy of maintenance treatment	AE	Relapse
Fan et al., 2022 (60)	31/F	PNP	Castleman’s disease	Prednisolone, cyclosporine, azithromycin	Ruxolitinib 5mg/d, prednisolone 10mg/d, cyclosporine 100mg/d	The symptoms were improved.	NA	Clinical presentation and laboratory tests improved.	NA	None
Gresham et al., 2023 (61)	74/F	DIBP	SCCHN, malignant melanoma	Prednisone, TCS	Upadacitinib, 15 mg/d	The patient demonstrate response to upadacitinib	NA	NA	NA	NA
Nash et al., 2023 (62)	81/F	BP	HT, dyslipidemia, osteoarthritis, endometriosis	Prednisone	Upadacitinib, 15 mg/d, prednisone	Complete resolution of disease.	NA	Continued efficacy with further healing of the skin and complete resolution of the disease.	None	None

AIBD, autoimmune bullous disease; AE, adverse effect; PNP, paraneoplastic pemphigus; DIBP, drug-induced bullous pemphigoid; BP, bullous pemphigoid; SCCHN, squamous cell carcinoma of the head and neck; HT, hypertension; TCS, topical corticosteroids; M, male; F, female; NA, not available; mg/d, mg/day.

### 4.4 Upadacitinib

Upadacitinib is a selective small molecule that functions as a JAK1 inhibitor. It has been approved for treating several medical conditions, including Crohn’s disease, psoriatic arthritis, atopic dermatitis, ulcerative colitis, and rheumatoid arthritis. Regarding AIBDs, researchers have used upadacitinib to treat BP (**Table 4**).

Gresham et al. presented a 74-year-old woman with recurrent squamous cell carcinoma of the head and neck who developed drug-induced BP while undergoing immunotherapy with a novel immunoglobulin-like transcript 4 inhibitor (MK-4830) and pembrolizumab (61). As her condition worsened, the patient decided to transition to end-of-life care. Upadacitinib was used to manage the BP symptoms, and after four weeks of treatment, the patient responded well, suggesting the potential therapeutic efficacy of upadacitinib in BP. It is essential to emphasize that this case involved the use of upadacitinib under exceptional circumstances to provide palliative care for skin symptoms in a patient receiving end-of-life care for metastatic cancer.

In a case study presented by Nash et al., an 81-year-old woman with BP showed an incomplete response to prednisone but achieved complete resolution with upadacitinib (62). BP is commonly observed in elderly patients who have multiple systemic comorbidities; therefore, it is essential to investigate the use of safer drugs for such individuals. Conducting further research on JAK inhibitors like upadacitinib would be useful in expanding the treatment options for this condition.

Kim et al. reported a case study of a patient suffering from EBP. The patient experienced intense itching and had widespread lesions. Treatment with upadacitinib led to a substantial reduction in itching and lesions without any adverse effects (63).

## 5 Systematic management of AIBDs

AIBDs are chronic conditions that can persist for several years or a lifetime with a high likelihood of relapse. The primary aim of treatment is to facilitate the healing of bullous and erosive cutaneous and/or mucous lesions, with the additional objectives of reducing pruritus, preventing or minimizing the recurrence of blistering eruptions, enhancing the quality of life of patients, and promptly identifying serious adverse effects associated with long-term therapy, especially in the elderly. Glucocorticoids and immunosuppressants are still the main treatment agents for AIBDs. Furthermore, the emergence of monoclonal antibody drugs has provided more options for the treatment of AIBDs. However, in clinical practice, doctors may still encounter patients for whom none of these treatments work. Therefore, discovering newer, safer, and more effective drugs has become a future trend in AIBDs therapy. JAK inhibitors interfere with the function of various inflammatory cytokines by targeting the JAK/STAT pathway, and thus, they have become new therapeutic agents for AIBDs. However, until now, only individual cases treated with JAK inhibitors have been reported, and no large-scale prospective clinical studies have been conducted to prove their efficacy. Based on the case reports available, JAK inhibitors are highly effective in patients who have failed to respond to treatment with glucocorticoids, immunosuppressive agents, and biologics. Therefore, it appears that, in general, JAK inhibitors are an attractive option for the treatment of AIBDs.

In addition to effectiveness, safety is also a significant concern regarding the current treatment options for AIBDs. The adverse effects of glucocorticoids include skin atrophy, fat redistribution, acne, weight gain, impaired glucose tolerance, insomnia, secondary infections, and femoral head necrosis (64). Conventional immunosuppressants may have side effects on liver and kidney function, and the potential consequences of systemic immunosuppression cannot be overlooked either. In recent years, advancements in immunological research have led to the development of new immunosuppressants, with rituximab being a representative example for treating AIBDs. However, it could lead to serious infections resulting from immunosuppression, and the invasiveness of its intravenous route of administration may also carry additional risks (65). Although JAK inhibitors have shown promising efficacy in clinical reports, their safety issues should not be ignored. For instance, one of the serious adverse effects of most JAK inhibitors is the activation of infections, including tuberculosis, herpes zoster, and hepatitis B (66). In addition, JAK inhibitors are associated with the risk of malignant tumors, such as lymphoma, which may be induced with the use of baricitinib, especially in patients with cancer (67).

Furthermore, attention should be paid to vascular events and cardiovascular risks, as several studies have shown that JAK inhibitors may cause thrombosis and platelet loss (68). Since the FDA added a black box warning to tofacitinib in 2019 and warned about the safety of JAK inhibitors, there has been ongoing controversy over cardiovascular events and thrombosis caused by JAK inhibitors. Some *post-hoc* analyses have shown that certain cardiovascular events and thrombosis are attributable to the use of baricitinib and tofacitinib (69, 70), but multiple meta-analyses have failed to confirm that the use of JAK inhibitors increases the risk of cardiovascular events (71, 72). Large, multi-center clinical studies of two highly selective JAK1 inhibitors (abrocitinib and upadacitinib) have also shown a very high safety profile, with no reported increase in cardiovascular events or higher thromboembolic risk (73, 74). This seems to imply that highly selective JAK inhibitors may have a lower cardiovascular risk. However, studies of abrocitinib and upadacitinib have mostly been conducted in patients with AD, who have a lower median age and have not been followed for a long duration.

In general, the safety of JAK inhibitors is a matter of debate. Considering that BP patients are predominantly elderly and prone to cardiovascular and metabolic diseases (1), the pros and cons of JAK inhibitors need to be carefully weighed before treatment. However, it is essential to note that the mechanisms by which JAK inhibitors cause cardiovascular events and thromboembolism risk are still unknown, so all patients should be educated and monitored. Despite this, based on current case reports, there have been no instances of severe adverse reactions to JAK inhibitor treatment for AIBDs. However, it cannot be ruled out that patients with severe adverse reactions have not been reported. Due to the potential adverse effects of JAK inhibitors, it is crucial to screen patients before administering them and to closely monitor them during treatment. Additionally, timely management of any adverse effects that may occur is vital when using JAK inhibitors.

## 6 Conclusions

We have reviewed the literature on the effectiveness of several JAK inhibitors in the treatment of AIBDs, including PV, PNP, BP, MMP, EBA, LPP, and DH. Based on the reported findings, we recommend careful monitoring, screening, and management of adverse effects on patients during the treatment of AIBDs with these agents. Regarding the gaps in the literature, the specific mechanisms of JAK/STAT in the pathogenesis and progression of AIBDs remain unclear, and more experimental studies are needed to further explore the role of this pathway in this group of diseases. Moreover, no clinical trials have been conducted to test the efficacy and safety of JAK inhibitors in the treatment of AIBDs. Larger and higher-quality long-term follow-up studies are essential to determine the efficacy and safety of JAK inhibitors in treating AIBDs. However, it must be mentioned that this review is a narrative review rather than a systematic review, and due to the scarcity of studies and lack of reports in the relevant fields, the conclusions are largely dependent on theoretical hypotheses and case reports. Therefore, the clinical use of JAK inhibitors in AIBDs should be concluded after joint discussions with patients.

## Author contributions

DH: writing – original draft. YZ: writing – original draft. LK: writing – original draft. JL: resources, methodology, writing – review & editing. YS: writing – review & editing. All authors contributed to the article and approved to the published version of the manuscript.
